# Treatment for Azoturia

**Published:** 1895-02

**Authors:** Louis Olry Lusson

**Affiliations:** Ardmore, Pa.


### REPORTS OF CASES.

### TREATMENT FOR AZOTURIA.

BY LOUIS OLRY LUSSON, ARDMORE, PA.

If called within the first two hours of this most dreaded malady, I can have my patient on his feet in just thirty-six hours. I first do the most important part, procure a warm sheepskin. If I cannot find a butcher handy I send the owner after a sheep and kill and strip it myself, placing the steaming hide over the kidneys and croup (right side down). Next catheterize; then aloes barb. ʒjss, and if in severe pain chloral hydrat. ʒj and the following:

| | | |
|---|---|---|
| ℞—Ext. Buchu . . . . . | fld. | |
| Ext. Juniperus Becca . . . | fld. | |
| Ammon. Acetas . . . | āā ℥iv. | M. |

Sig.—Two (2) tablespoonfuls (℥j) in the same quantity of water *every hour* for twenty-four hours, then every two hours for the next twelve hours.

Method of Treating Cases of Azoturia.—I make a practice to catheterize every eight hours and administer chloral in ʒj doses when patient is very uneasy, allowing water ad libitum, and turn every few hours. When using sheepskin (fresh) in conjunction with drugs above described I rarely lose a case. I do not offer this as a new mode of treatment, but simply as the result of my personal experience.

